# Prevalence and Risk Factors for Antenatal Depression in Ethiopia: Systematic Review

**DOI:** 10.1155/2018/3649269

**Published:** 2018-07-09

**Authors:** Wondale Getinet, Tadele Amare, Berhanu Boru, Shegaye Shumet, Wubet Worku, Telake Azale

**Affiliations:** ^1^Department of Psychiatry, College of Medicine and Health Science, University of Gondar, Gondar, Ethiopia; ^2^School of Nursing, College of Medicine and Health Science, University of Gondar, Gondar, Ethiopia; ^3^Public Health Institute, College of Medicine and Health Science, University of Gondar, Gondar, Ethiopia

## Abstract

**Introduction:**

Antenatal depression is a syndrome, in which women experience depressed mood, excessive anxiety, insomnia, and change in weight during the period of pregnancy. Maternal depression negatively influences child outcomes and maternal health. Antenatal depression was measured by different rating scales, namely, BDI, EPDS, and PHQ. The objective of this systematic review was to synthesize logical evidence about the prevalence and potential risk factors of antenatal depression in Ethiopia.

**Methods:**

Our team explored multiple databases including PSYCHINFO, MEDLINE, Embase, Google Scholar, and Google Search to detect studies published with data on the prevalence of antenatal depression. We found 246 research papers on antenatal depression, of which 210 did not correspond to the title and 27 were duplicates. Subsequently, nine articles were used for synthesis prevalence, of which four studies were selected in the analysis of the effect of unplanned pregnancy on antenatal depression. Figures were extracted from published reports and grey literature, and any lost information was requested from investigators. Estimates were pooled using random-effects meta-analyses.

**Results:**

The pooled prevalence of antenatal depression for five studies selected, which had used BDI, was 25.33 (20.74, 29.92). The other four studies that had included other screening tools (3 EPDS and 1 PHQ) had the prevalence decreased to 23.56 (19.04, 28.07), and the pooled effect of unplanned pregnancy on antenatal depression was 1.93 (1.81, 2.06). Factors such as age, marital status, income, occupation, history of the previous mental disorder, antenatal follow-up, unplanned pregnancy, complication during to pregnancy, age of mother during pregnancy, conflict, and social support were associated with antenatal depression.

**Conclusions:**

Antenatal depression is a common maternal problem; further attention should be given to the effect of unplanned pregnancy, social support, pregnancy-related complications, family conflicts, and violence on pregnant women. All these are possible risk factors for antenatal depression.

## 1. Background

A global report by World Health Organization indicated that neuropsychiatric disorders represent a total of 28 % of the diseases of which more than one-third are caused by depression [1]. Mental illness like depression, a cause of almost 12% of total years lived with disability worldwide [2], causes physical and psychological pain for both patients and caregivers [3]. Predictions show that depression becomes the second leading cause of disease burden by the year 2020 [4] and the leading cause of disability among women in childbearing age group, yet it does not remain a part of reproductive healthcare [5].

Pregnancy-related depression is the one encountered during the time of child conception. It is manifested by persistent sadness and associated with additional somatic symptoms like decreased energy, sleep disturbance, weight loss, hopelessness, difficulty to think, poor concentration, disturbed sleep, and trouble with appetite along with inability to feel happiness [6, 7].

Studies done in different countries showed that untreated antenatal depression in pregnant women has consequences on fetus like increased risk of preterm birth (PTB), low birth weight (LBW), intrauterine growth restriction (IUGR) [8], neurobehavioral development and adverse perinatal outcome like lower birth weight, childhood behavioral disturbance, difficulty in language development, unhealthy maternal behaviors, maternal impairment during the postpartum period, substance abuse, and decrease in parenting quality and effectiveness [6, 8–13].

Antenatal depression affects about 1/10 women in high-income countries [29, 30]. It is a common public health problem in sub-Saharan Africa ranging from 8% to 40% [24, 31–33]. Many epidemiological findings worldwide have reported that depressive symptoms are more common during pregnancy than during the postpartum period [34–37]. Depressive disorders after giving birth have a strong association with the depressive disorder during pregnancy [38–42].

Factors which can predispose women to antenatal depression are almost similar in different countries like unplanned pregnancy, physical health, financial situation, social life, educational status, poor antenatal follow-up, malnutrition, pregnancy-related complications, past history of mental disorder, and partner violence [18, 24, 25, 43].

Antenatal depression among Brazilian women indicated that the prevalence of depression in the third trimester of pregnancy was 38% and after delivery in the first six months was 43% [14]. The percentages of antenatal depression varied in other countries and regions of the world as indicated in Table 1.

The prevalence of antenatal depression in Ethiopia is different from one town to another; it ranges between 11.8% and 31.2 percent with almost 25% in Addis Ababa. The percentage in the rest of the towns in Ethiopia are: 23% in Gondar, 25.6% in Shashemene, 28.7% in Sodo district, 31.1% in Maichew, 31.2% in Adama, 11.8% in Debretabor, 19.9% in Gilgel Gibe, and 17.9% in Afar Dubti [28] (Table 1).

However, using different methodology and psychometric tools, an extensive variation was found among the prevalence rates of antenatal depression in Ethiopia as described by different investigators at different places and time. Such limited and discrepant logical information about antenatal depression in our country resulting from its disparity and backgrounds had not been systematically reviewed by health decision makers and implementers. Somewhat, until now no review has shown how to find target data in relation to pregnancy, socioeconomic, and demographic factors and to catch out the risk factors of antenatal depression.

The objective of this systematic review and meta-analysis was to make scientific evidence on the prevalence of antenatal depression and associated factors in Ethiopia. Is there an association between unplanned pregnancy and antenatal depression? Result of this study would benefit health organizers who have interest in formulating intervention strategies for antenatal depression in the native context.

## 2. Methods

### 2.1. Search Strategy

The protocol and writing of the results of this systematic review and meta-analysis were based on PRISMA guidelines [44]. A comprehensive literature search was done by entering the following titles in PubMed, MEDLINE, Cochrane Library, Embase, Google Scholar, and Google Search: “prevalence of antenatal depression in Ethiopia”, “associated factors of antenatal depression in Ethiopia”, “maternal depression in Ethiopia”, and “pregnancy-related depression in Ethiopia”. The Cochrane Library review database was also searched by the terms “prevalence of antenatal depression in Ethiopia” and “associated factors of antenatal depression in Ethiopia”. Finally, we used MeSH terms and All terms words in MEDLINE and keywords in Embase. The titles and abstracts of all identified citations were screened for relevance and the full text of potentially relevant articles was obtained and assessed for eligibility. No reviews could be found from Ethiopia.

### 2.2. Inclusion and Exclusion Criteria

As English is the language used in Ethiopia in the media and in higher institutions, articles not written in English were excluded from our study. In addition, articles which were not conducted in Ethiopia were also excluded. All publications of all years on the prevalence and associated factors of antenatal depression in Ethiopia were included in the study.

### 2.3. Critical Appraisal, Data Extraction, and Synthesis

The potentials of each systematic review were assessed by using a checklist adjusted from Joanna Briggs Institute (JBI) Critical Appraisal for Study Papers [45]. The checklist focuses on the following criteria: clearness of statement of objectives, the appropriateness of methodology, the appropriateness of research design, justification of selection strategy detail, appropriateness of data collection methods, the relationship between the researcher and the participant, consistency of data analysis, pure statement of findings, and discussion of the value of the research. The assessment of each of the studies in accordance with the checklist revealed that almost all of the reports were of acceptable quality. Data were primarily appraised for quality and then extraction was made using data extraction method. Data were analyzed using STATA V.14 statistical software. Due to the possibility of heterogeneity among the studies, random-effects meta-analysis models were preferentially reported. We developed the data extraction form that fits the specific objective of the systematic review. It included date of publication, name of the author, objective of the study, setting, study methods, and results. The data was initially assembled into themes like the prevalence of antenatal depression and associated factors. Meta-Analysis software was used to compute the pooled prevalence for the first five studies which used the same diagnostic tool Beck Depression Inventory (BDI) [46–50], the three which used Edinburgh Postnatal Depression Scale (EPDS) [28, 51, 52], and the one which used Patient Health Questionnaire (PHQ) [53]. Lastly, additional prevalence number was computed for the nine studies irrespective of the type of measuring tool they used for screening. At that time the report was made based on the themes.

### 2.4. Search Outcomes

**Figure 1:**
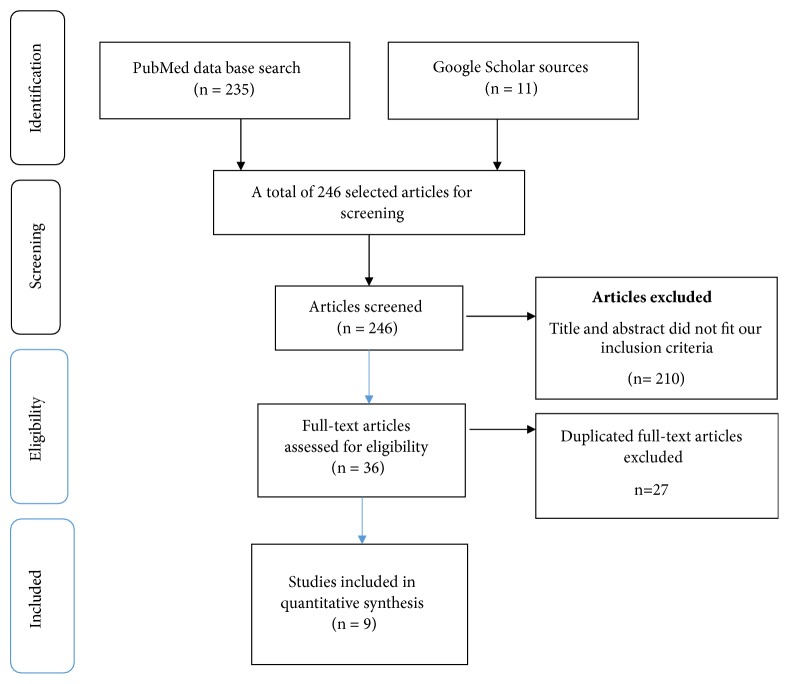
Flow chart of study selection.

The electronic searching of literature produced 246 articles: 210 did not fit the title, the abstract, and our inclusion criteria, and 27 were duplicates. Finally, nine articles were used for quantitative synthesis, four of which included the effect of unplanned pregnancy on antenatal depression (Figure 1).

### 2.5. Study Area and Settings

All studies were conducted in Ethiopia. There were no time restrictions during the database search. Seven of the studies were cross-sectional surveys and two were cohort study. No randomized controlled trials and case controls were obtained (Table 2).

## 3. Results

### 3.1. Antenatal Depression

**Figure 2:**
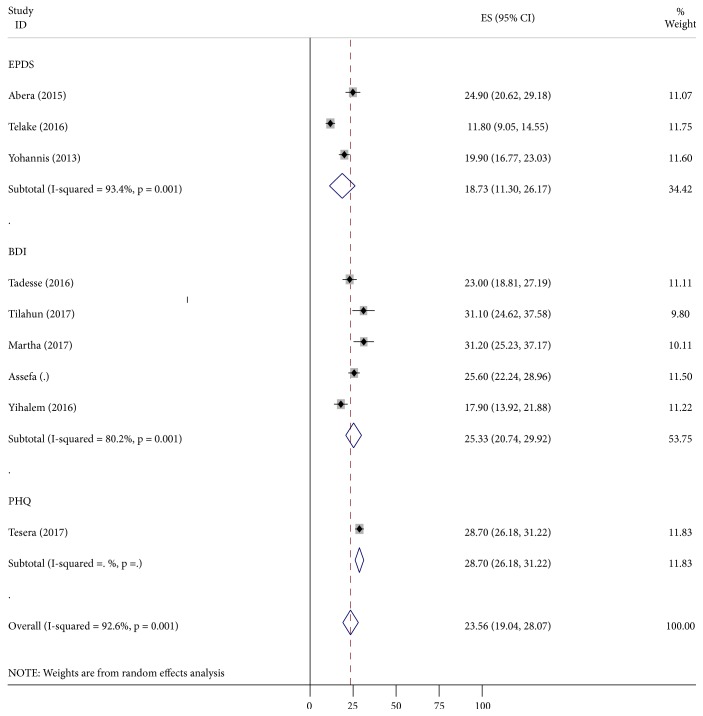


Magnitude of antenatal depression in Ethiopia is between 11.8% and 31% [48, 53, 54]. However, pooled prevalence of antenatal depression of five studies measured by BDI was 25.33 (20.74, 29.92) as shown in Figure 2. Still, pooled prevalence for the other three studies which used EPDS was 18.73 (11.30, 26.17). The prevalence rates in the nine studies used 5 BDI, 3 EPDS, and 1 PHQ. Pooled together, the prevalence was 23.56 (19.04, 28.07) (Figure 2).

### 3.2. Publication Bias

**Figure 3:**
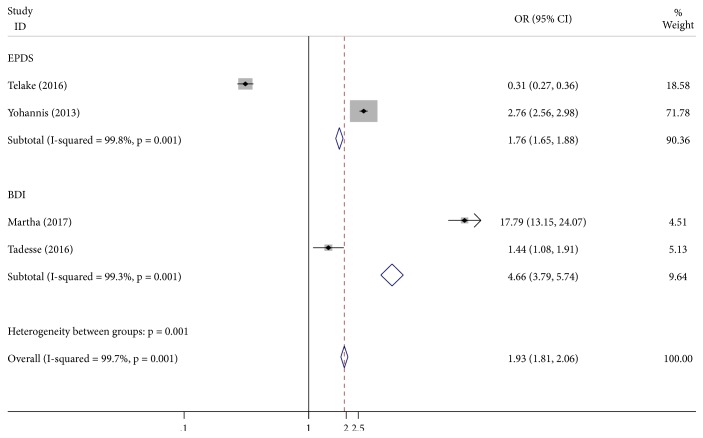
Pooled effect of unplanned pregnancy on antenatal depression in Ethiopia.

There was no evidence of bias upon inspection of funnel plots. However, due to the restricted number of studies included in the analysis, Egger's linear regression model was also used. The Egger's regression test for asymmetry suggested that there was no significant publication bias for the prevalence of antenatal depression and effect of unplanned pregnancy *p* = 0.42, *p* = 0.92, respectively (Figures 2 and 3).

The prevalence of depression was high among pregnant women particularly in sub-Saharan Africa [24, 31–33]. All researchers tried to assess factors that lead pregnant women to antenatal depression (Tables 3 and 4). Concerning marital status women who were unmarried were more likely to be depressed than married mothers [47, 48]. However, women with age range between 20 and 29 were less likely to develop antenatal depression than women with age range between 14 and 19 [46]. Also, housewives compared to women who were concerned in government employment and private jobs were more likely to be depressed [46, 48]; additionally, merchant and daily laborers were at higher odds than government employees [46].

Pregnant women with a low income (monthly income of <1500 Eth. Birr) were more vulnerable to depression [48]; those with an income of above 1000 Eth. Birr were less likely to develop antenatal depression [47]. Moreover, women with money issues and debt have higher odds of depression [49, 54] (Table 3).

Women who had previous history of diagnosed depression were at higher odds of antenatal depression compared to women with no history of previous depression [28, 54]. In more than half of the reviewed papers, pregnant women who had not planned their current pregnancy were more likely to have antenatal depression than those who had planned their pregnancy [28, 49–51, 54]. Pregnant women with irregular antenatal follow-up or no previous antenatal follow-up were at higher odds to develop depression [46]. Moreover, pregnant mothers with history of obstetric complications were more likely to develop antenatal depression in the majority of the reviewed papers (Table 4) compared to those with no previous history of obstetric complications [47]. Women who self-reported their complications during pregnancy [53], those who had the fear of complicated pregnancies [49], those with current complicated pregnancies [54], and those with history of abortion or stillbirth [49, 54] had all the predispositions to develop antenatal depression.

Women in third trimester of pregnancy have high risk to develop maternal depression [54]. Other factors like partner violence [47] and marital conflict [47, 49, 50] could cause antenatal depression. Social support helped pregnant women have less risk, while absence of social support contributed to the development of antenatal depression [28, 50, 51] (Table 4).

## 4. Discussion

The aim of this systematic review and meta-analysis is to assess the effect of unplanned pregnancy and associated factors on the prevalence of antenatal depression. Diverse proportions of prevalence have been stated through different investigators, yet pooled prevalence of antenatal depression of nine studies was 23.56 (19.04, 28.07). It was similar to the prevalence reported in a cross-sectional study which was conducted in Gondar [46] and similar to study done in Addis Ababa and Shashemene [28, 47]. The pooled prevalence of antenatal depression suited the highest, i.e., 25.33 (20.74, 29.92), once BDI was used for screening, 28.7(19.04, 29.92) when PHQ was used, and 18.73 (11.30, 26.17) when EPDS was used. Psychometric tool used for measuring antenatal depression and cutoff point used to label the mother's depressive disorder are different from those used in the current review. Ahead of that, the study time and stage of pregnancy at which the data was collected could be considered as a source of expected variation between the current review and compared studies; the nature of psychometric tools used like BDI is liable to somatic symptoms when women are exposed to physical symptoms; when there is medical comorbidity, it may increase the value of depression with contrast to others.

The pooled prevalence of antenatal depression in nine studies (23%) was also the same as the prevalence of antenatal depression in Asian and African countries like Oman and Nigeria [19, 22], respectively. The prevalence was similar to that of the global figure and represents the prevalence in the whole country as the samples were taken from various parts of Ethiopia. On the one hand, the pooled prevalence of antenatal depression in Ethiopia was lower than those in studies conducted in different countries like Brazil [14], America [20], Ukraine [21], South Africa [23], Cape Town [24], Tanzania [25], Sodo in Ethiopia [53], Maichew in Ethiopia [48], and Adama in Ethiopia [49]. On the other hand, the prevalence was higher than those in studies done in Sri Lanka [15], Italy [16], Mumbai [17], Bengaluru [18], Ghana [26], Malawi [27], Debretabor in Ethiopia [54], Gilgel Gibe in Ethiopia [51], and Afar Dubti in Ethiopia [50].

Antenatal depression was considerably higher among women who did not plan their current pregnancy. In almost more than half of the papers reviewed, unplanned or unwanted pregnancy was the factor leading to antenatal depression compared to planned pregnancy [28, 49–51, 54]. The pooled effect of unplanned pregnancy on antenatal depression was 1.93 (1.81, 2.06). Unplanned pregnancy might have some influence on depression causing physical, psychological, and social changes in women.

The other factor that had a significant association with maternal depression was marital status of women. Unmarried women have a higher rate of prevalence for antenatal depression than married ones [47, 48]. The reason could be that unmarried women might practice more loneliness, poorer social support, and lower self-confidence and are more likely to be living alone, which are usually regarded as risk factors for depression in the pregnant women.

In our review, monthly family income of pregnant mothers was an important variable that holds huge share in antenatal depression [47–49, 54].This indicated that pregnant mothers with low income, with debt, and with economic problems are more likely to develop antenatal depression than those who have no debt with medium and high monthly domestic income. The reason could be that low income decreases the likelihood of independent living and increases the psychosocial stress of life in pregnant women, the worry of future mother about the newborn baby, and the financial struggle they might encounter after the birth.

As previously described, women occupation was significantly associated with antenatal depression, for example, nongovernmental employment, being merchant or daily laborer, and being a housewife [46, 48]. The fear of being financially dependent on others or financially not sufficient could be the reason for the potential to develop antenatal depression. Relationship between socioeconomic factors and antenatal depression requires additional research; it contradicts the findings in developed nations where the prevalence of depression was comparatively higher in high-income countries than in developing countries.

Women with previous history of depression were more likely to develop depression during pregnancy. Those women might be more biologically vulnerable due to the hormonal changes during pregnancy. The hormonal changes together with their psychological and social situation will increase their susceptibility to depression development and exposure to recurrent depression [28, 54]. Previous history of obstetric complications was reported to be a risk factor for developing antenatal depression [47, 49, 53, 54]. It might be a psychological fear of having another complication in the current pregnancy. Conflict with husband and partner violence are other risk factors causing antenatal depression [47, 49, 50]. Other factors such as age, antenatal follow-up, age of pregnancy, and social support have consistently an impact on antenatal depression. These results were similar with studies carried out in the developed countries. The results of the review suggest common risk factors of developing antenatal depression worldwide. Antenatal depression is present in most countries irrespective of the ethnic origin. The prevalence of antenatal depression in Ethiopia is comparatively higher than other countries. This result has effects for policy makers, health administrators, professionals, and the community. This finding provides evidence on prevalence of antenatal depression on Ethiopian women with locally relevant data, where antenatal depression may negatively influence mother-infant health outcomes. Actions to reduce the effect of antenatal depression should be taken, and these priority areas must be considered: awareness, family contribution, prevention and early intervention, and support of the primary healthcare system. Intervention studies that evaluate current and previous policy initiatives and the wider impacts of health system strengthening on antenatal depression and outcomes should be a focused priority.

National intervention plans should focus on resolving conflicts and intimate partner violence, giving social support, and counseling on the age of women to get pregnant and their marital status. These are the key risk factors leading to antenatal depression. However, due to shortage of available and indexed articles about antenatal depression in Ethiopia, the review was conducted on only the current limited literature. Therefore, the prevalence of antenatal depression reported here was an estimation rather than an exact number.

## 5. Conclusions

Antenatal depression is a common maternal and public health problem; further attention should be given to unplanned pregnancy, social support, pregnancy-related complications, conflicts, and household violence. A proper obstetric and maternal care in women as well as a routine screening of women in the antenatal period may decrease the prevalence of depression during pregnancy.

## Figures and Tables

**Table 1:** Prevalence of antenatal depression in different parts of the world.

**Country or town**	**Percentage of antenatal depression**	**Reference**
Brazil	38%	[14]

Sri Lanka	16.2%	[15]

Italy	15%	[16]

Mumbai	9.2%	[17]

India Bengaluru	12.3%	[18]

Oman	24.3%	[19]

African American women (In low income category)	59%	[20]

Ukraine (HIV positive women)	27%	[21]

Nigeria	8.3%,	[22]

Abeokuta Nigeria	24.5%	[22]

South Africa	47%	[23]

Cape Town	39%	[24]

Tanzania	38.8%	[25]

Ghana	10%	[26]

Malawi	11.6%	[27]

Addis Ababa	25%	[28]

**Table 2:** Prevalence and associated factors of antenatal depression in Ethiopia.

Author	Year	Study area	Focus of study (objective)	Methods	**Tools**	**Cut-off**	%	Main finding
Abera et al.	2015	Addis Ababa	Prevalence of antenatal depression and associated factors among pregnant women	Cross sectional	**EPDS**	**13**	24.9	(i) Previous history of depression is 3 times at higher odds (ii) Unplanned pregnancy is 3 times at higher odds (iii) Lack of father support is 2 times higher odds

Tadesse et al.	2016	Gondar	Prevalence and Associated Factors of Antenatal Depression among Women Attending Antenatal Care Service	Cross sectional	**BDI**	**16**	23	(i) Female aged between 20 and 29 have 0.18 higher odds (ii) Housewives are 2.5 times at higher odds (iii) Merchant and daily laborers are 3.4 times at higher odds (iv) Previous antenatal follow-up pattern irregular is 11.4x at higher odds (v) No previous ANC follow-up pattern is 11.9x at higher odds

Assefa GW	2015	Shashemane	Prevalence and factors associated with antenatal depression among women following antenatal care	Cross sectional	**EPDS**	**13**	25.6	(i) Unmarried female are 3x at higher odds (ii) Female who did not have negative obstetric history were less likely to have depressive symptom 0.77x (iii) Female with monthly income of above 1000 Eth were less likely to have depression (0.22X less) than female with income less than 500 Eth. (iv) Conflict with husband (0.35x) (v) Lack of support (0.35x) (vi) History of intimate partner violence (0.19x)

Tesera et al.	2017	Sodo	Antenatal depressive symptoms and perinatal complications	Cohort	**PHQ-9**	**5**	28.7	(i) Self-reported complications in pregnancy are 2x at higher odds (ii) 1.8x complicated labor (iii) 1.7x postpartum complication

Tilahun et al.	2017	Maichew	Prevalence of Antenatal Depressive Symptoms and Associated Factors among Pregnant Women	Cross sectional	**BDI**	**14**	31.1	(i) Low level of income is 5.12x at higher odds (ii) Unmarried women are 4x at higher odds (iii) Housewives are 4.2x at higher odds

Martha et al.	2017	Adama	Prevalence and predictors of antenatal depressive symptoms among women attending Adama Hospital	Cross sectional	**BDI**	**21**	31.2	(i) Previous abortion is 2.86x at higher odds (ii) Fear of pregnancy complications is 3.49X at higher odds (iii) Economic problems are 9.52x at higher odds (iv) Unwanted pregnancy is 6.99x at higher odds (v) Marital conflict is 22.68x at higher odds

Telake et al.	2017	Debretabor	Prevalence and Predictors of Depression among Pregnant Women	Cross sectional	**EPDS**	**12**	11.8	(i) Having debt is 2.79x at higher odds (ii) Unplanned pregnancy is 2.39x at higher odds (iii) History of stillbirth is 3.97x at higher odds (iv) History of abortion is 2.57x at higher odds (v) Third trimester of pregnancy is 1.7x at higher odds (vi) Complication in the current pregnancy is 3.29x at higher odds (vii) Previous history of depression is 3.48x at higher odds

Yohannes et al.	2013	Gilgel Gibe	The association of unwanted pregnancy and social support with depressive symptoms in pregnancy	Cohort	**EPDS**	**13**	19.9	(i) Unwanted pregnancy is 1.96x at higher odds (ii) Social support during pregnancy moderate 0.27 high 0.23

Yihalem et al.	2016	Afar Dubti	Factors Associated with Antenatal Depression among Pregnant Women in pastorals areas	Cross sectional	**BDI**	**17**	17.9	(i) Marital conflict is 6.45x at higher odds (ii) Planned pregnancy is 0.04 at higher odds (iii) Medium social support is 0.21x at higher odds

**Table 3:** Demographic risk factors of antenatal depression.

**Demographic Factors**	**Risks of demographic factors on antenatal depression**	**Author**
Marital Status	Unmarried women have 4 times higher risk than married women	Tilahun et al., 2017
	Single women are 3x at higher odds than counter parts	Assefa GW, 2015

Age	Women aged between 20 and 29 were 72% times less likely to develop antenatal depression than those aged between 14 and 19	Tadesse et al., 2016

Occupation	Housewives are 2.5x more likely to have antenatal depression than government employees	Tadesse et al., 2016
	Merchant and daily laborers are 3.4x at higher odds than government employees	Tadesse et al., 2016
	Housewives are 4.2x at higher odds than private workers	Tilahun et al., 2017

Income	Those with monthly income of above 1000 Eth. Birr are 78% less likely to develop antenatal depression compared to those with income below 500 Eth. Birr	Assefa GW, 2015
	Income less than 1500 Eth. Birr is 5.12x at higher odds to develop antenatal depression than income >1500 Eth. Birr.	Tilahun et al., 2017
	Economic problems are 9.52x at higher odds to develop antenatal depression	Martha et al., 2017
	Having debt was 2.79x at higher odds to develop antenatal depression	Telake et al., 2017

**Table 4:** Maternal risk factors for antenatal depression.

**Maternal Factor**	**Likelihood to develop antenatal depression**	**Author**
Mental disorder	Previous history of depression is 3x and 3.48x at higher odds than no history of depression	Abera et al., 2015; Telake et al., 2017

Pregnancy related	Unplanned pregnancy is 3x and 2.39 at higher odds than planned pregnancy	Abera et al., 2015; Telake et al., 2017
	Unwanted pregnancy is 6.99x and 1.96x at higher odds than wanted pregnancy	Martha et al., 2017; Yohannis et al., 2013
	Women with planned pregnancy are 96% less likely to report depression than those with unplanned pregnancy	Yihalem et al., 2016

ANC follow-up	Previous irregular ANC follow-up is 11.4x at higher odds than regular ANC follow-up	Tadesse et al., 2016
	No previous ANC follow-up is 11.9x at higher odds than those with ANC follow-up.	Tadesse et al., 2016

Pregnancy related complications	Women with no previous history of obstetric complications are 23% less likely to have depressive symptoms.	Assefa GW, 2015
	Self-reported complications in pregnancy are 2x at higher odds	Tesera et al., 2017
	Fear of pregnancy complications is 3.49X at higher odds of depression	Martha et al., 2017
	Complication in the current pregnancy is 3.29x at higher odds of depression than absence of complication	Telake et al., 2017
	History of previous abortion is 2.86x at higher odds	Martha et al., 2017
	History of stillbirth is 3.97x at higher odds	Telake et al., 2017
	History of abortion is 2.57x at higher odds	Telake et al., 2017

Trimester	Pregnancies in the third trimester are 1.7x at higher odds than first trimester	Telake et al., 2017

Conflicts	Presence of conflicts with husband is 0.35x at higher odds to develop antenatal depression	Assefa GW,2015
	Presence of marital conflicts is 22.68x and 6.45x at higher odds to develop antenatal depression	Martha et al., 2017; Yihalem et al., 2016
	History of intimate partner violence is 0.19x at higher odds to develop antenatal depression	Assefa GW,2015

Social support	Lack of father support is 2x at higher odds to develop antenatal depression	Abera et al., 2015
	Lack of support is 0.35x at higher odds to develop antenatal depression	Assefa GW, 2015
	Women with moderate, medium and high social support during pregnancy are less likely to report depressive symptoms (0.27x odds, 0.21x odds and 0.23x odds respectively)	Yohannis et al., 2013

## Abbreviations

ANC: Antenatal care
BDI: Beck depression inventory
EPDS: Edinburgh postnatal depression scale
IUGR: Intrauterine growth restriction
LBW: Lower birth weight
PTB: Preterm birth
PHQ: Patient health questionnaire
WHO: World Health Organization.
